# Inositol Polyphosphate-5-Phosphatase F (INPP5F) inhibits STAT3 activity and suppresses gliomas tumorigenicity

**DOI:** 10.1038/srep07330

**Published:** 2014-12-05

**Authors:** Hong Sug Kim, Aiguo Li, Susie Ahn, Hua Song, Wei Zhang

**Affiliations:** 1 Neuro-Oncology Branch, National Cancer Institute and National Institutes of Neurological Disorders & Stroke, National Institutes of Health, Bethesda, MD 20892, USA

## Abstract

Glioblastoma (GBM), the most common type of primary malignant brain tumors harboring a subpopulation of stem-like cells (GSCs), is a fast-growing and often fatal tumor. Signal Transducer and Activator of Transcription 3 (STAT3) is one of the major signaling pathways in GSCs maintenance but the molecular mechanisms underlying STAT3 deregulation in GSCs are poorly defined. Here, we demonstrate that Inositol Polyphosphate-5-Phosphatase F (INPP5F), one of the polyphosphoinositide phosphatases, is differentially expressed in GSCs from glioma patients, and is identified as an inhibitor of STAT3 signaling via interaction with STAT3 and inhibition of its phosphorylation. Constitutively expressed INPP5F showed to suppress self-renewal and proliferation potentials of glioblastoma cells and reduced tumorigenicity of glioblastoma. In addition, loss of *INPP5F* gene in gliomas is significantly correlated with lower overall patient survivals. These findings suggest that INPP5F is a potential tumor suppressor in gliomas via inhibition of STAT3 pathway, and that deregulation of INPP5F may lead to contribution to gliomagenesis.

Despite advances in multidisciplinary therapies, glioblastomas still have an overall poor prognosis with a median survival of less than 16 months for treated patients^1^. Successfully identifying the pivotal molecular mediators of glioblastoma progression and tumor resistance is therefore critical to improve the outcome of glioblastoma treatment. glioblastomas represents striking cellular heterogeneity with highly altered chromosomes. Especially, chromosome 10q locus is frequently deleted in gliomas^2^. Located in chromosome 10q26.11, INPP5F (Inositol Polyphosphate-5-Phosphatase F) is identified to be one of the polyphosphoinositide phosphatases that regulate phosphatidylinositol 4, 5-bisphosphate (PtdIns [4, 5] P_2_ [PIP2]) and phosphatidylinositol 3, 4, 5-bisphosphate (PtdIns[3,4,5]P_3_ [PIP3])^3^. Recently it has been reported that *INPP5F* expression is regulated by Hdac2, resulting in modulation of the AKT-Gsk3β pathway in Hdac2 deficiency and transgenic mice^4^. INPP5F knockout (*INPP5F*^-/-^) mice increase PIP3 in hearts after IGF-1 treatments, and INPP5F regulates cardiac hypertropic responsiveness through PI3K/AKT pathway^5^. However, INPP5F functions is not fully understood and reported in cancer biology.

It has been reported that several signaling pathways are abnormally activated in gliomagenesis, such as phosphatidylinositol-3′-kinas (PI3K)/Akt, Mammalian target of rapamycin (mTOR), Ras/Extracellular signal regulated kinase (Erk), and Janus kinase (JAK)2/signal transducer and activator of transcription 3 (STAT3) signaling pathways. STAT3 belongs to a family of transcription factor (TFs) and plays central important role in cell growth^6^, and its activation has been described in 70% of human cancers^7^. Increased evidences show that STAT3 is an important and often essential factor in oncogenic cellular transformation and in cancer. It is previously reported that STAT3 is a required target of the Src oncoprotein, and expression of dominant-negative STAT3 blocks Src-induced cellular transformation^8^,^9^. In addition, constitutive activation of STAT3 has been reported in several primary cancers and in many oncogene-transformed cells, indicating a direct oncogenic role of STAT3^10^. STAT3 activation occurs following cytokine- or growth factor-receptor activation, which involves phosphorylation within the cytoplasm, dimerization and nuclear translocation^11^. Nuclear translocation of STAT3 requires nuclear localization signals (NLS), which are in the coiled-coil domain^12^ and dimer-dependent^13^.

To identify glioblastoma related genes and improve novel targeting therapeutics, many studies have been done to characterize gene alterations on glioma surgery specimens by gene expression and single nucleotide polymorphism (SNP) genotyping arrays and the profiles are stored and could be analyzed in databases such as TCGA (The Cancer Genome Atlas)^14^ and REMBRANDT (REpository of Molecular BRAin Neoplasia DaTa)^15^. Using multifaceted genomic and expression analysis approach with TCGA and REMBRANDT, we identified that *INPP5F* is frequently inactivated via chromosome deletion and mRNAs suppression in GBM. Based on this, we hypothesized that INPP5F might act as a tumor suppressor through regulation of oncogenic proteins by phosphorylation inhibition. Here, we showed that INPP5F regulates the STAT3 pathway via interaction and inhibition of STAT3 phosphorylation, and suppress gliomagenesis. Our data provide insight into the molecular underpinning of this potential tumor suppressor gene in glioblastomas.

## Results

### INPP5F is targeted for deletion and suppression in glioblastomas

**Figure 1:**
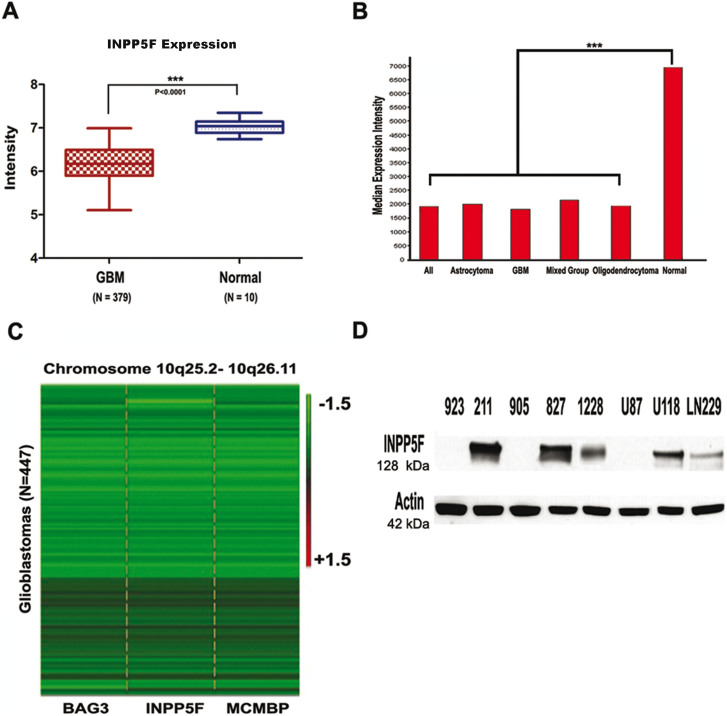
INPP5F gene locus is frequently deleted and the expression is deregulated in gliomas. (A) The box and whisker plots show INPP5F expression in 379 glioblastomas (dark red) and in 10 non-tumor brain samples (blue) analyzed from TCGA dataset. The box indicates the 25% ~ 75% quartile and the bars indicate the upper and lower extreme values within the groups (***: p <0.0001). (B) Median expression intensity of INPP5F (probe 203607) in REMBRANDT dataset represents that INPP5F expression in all types of gliomas is decreased compare to non-tumor brain samples (***: p<0.0001). (C) Copy number heatmap of INPP5F and its neighboring genes from TCGA glioblastomas dataset. The green color in the map indicates deletion and black color represents normal copy numbers. (D) INPP5F is differently expressed in GSCs and established glioblastoma cell lines at the protein level.

Based on the fact that chromosome 10q locus is frequently deleted in gliomas and INPP5F gene is located in Ch10q26.11, we first examined if *INPP5F* is one of these highly altered genes, by looking for naturally occurring alterations within the *INPP5F* locus that could affect INPP5F expression and/or activity in gliomas. We analyzed gene expression data from TCGA and REMBRANDT (Figure 1A and 1B). Compared with controls, INPP5F gene expression is significantly decreased in 379 glioblastoma samples in TCGA, and in 545 all types of gliomas in REMBRANDT (both p<0.0001). Next, we checked the copy number of *INPP5F* and adjacent genes' locus including *BAG3* (BCL2-associated athanogene 3) and *MCMBP* (Minichromosome Maintenance Complex Binding Protein). In 447 glioblastoma samples examined, 385 (86%) exhibited Ch10q25.2-10q26.11 deletion (log2 ratio less than -0.5) and 7 have shown *INPP5F* gene deletion only (Figure 1C). Furthermore, the INPP5F expression in patient-derived GSCs and glioblastoma cell lines were analyzed at protein levels and a differently expression pattern of INPP5F protein in these cell lines was observed (Figure 1D). These results that decreased expression of *INPP5F* and frequent deletions of Ch10q26.11 locus in gliomas strongly suggest that *INPP5F* is targeted for inactivation during glioma pathogenesis.

### INPP5F suppresses glioblstoma cell proliferation and self-renewal activity

**Figure 2:**
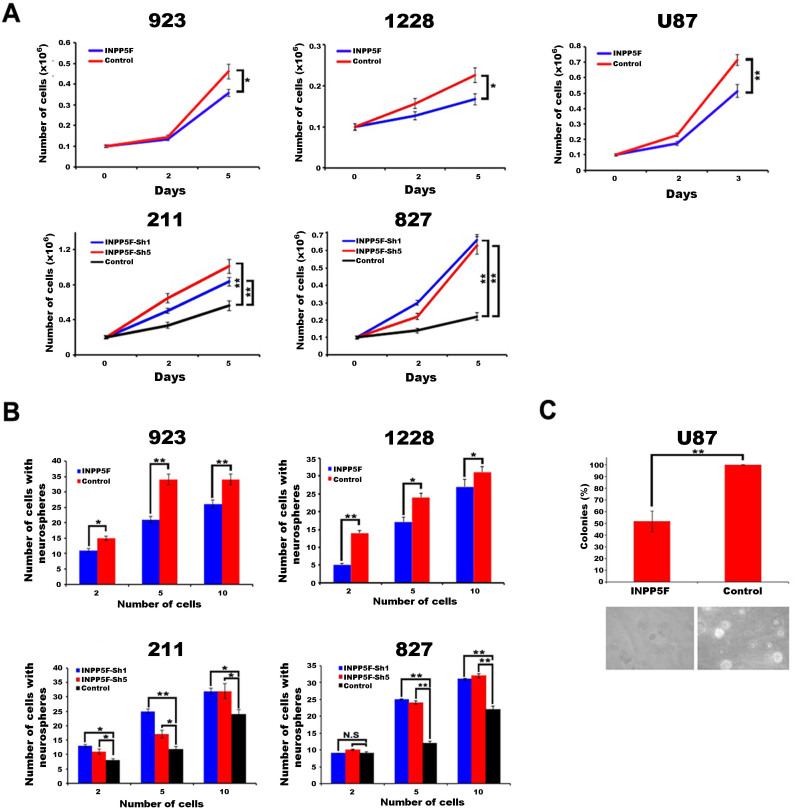
INPP5F regulates glioblastoma cell proliferation and self-renewal activities *in vitro*. (A) INPP5F overexpression decreased glioblastoma cell proliferation, while INPP5F knockdown increased proliferation activity. (B) Analysis of self-renewal activity represents that INPP5F overexpressing GSCs reduced sphere formation and INPP5F knockdown increased sphere forming activity. (C) INPP5F overexpression shows a decreased colony forming activity. (NS: no significant; * p<0.05; ** p<0.001; compared to control). Error bars represent standard deviation (S.D., experiment performed in triplicates).

Next, we generated constitutively INPP5F overexpressing and knockdown glioblastoma cell lines to investigate the biological role of INPP5F in gliomas tumorigenicity. As shown in Figure 2A, INPP5F overexpressing cells exhibited a decreased proliferation, and INPP5F knockdown cells enhanced cell proliferation activity compared to controls. Consistent with the proliferation activity, INPP5F overexpressing GSC cells exhibited a decreased self-renewal activity while the INPP5F knockdown GSC cells showed an increasing (Figure 2B). In addition, INPP5F overexpressing U87 cells showed significantly decreased anchorage independent cell growth activity (Figure 2C). These results demonstrate the inhibitory effects of INPP5F on glioblastoma cell activities *in vitro*, supporting that INPP5F might be a tumor suppressor in glioblastomas.

### INPP5F regulates STAT3 activation and pathway

**Figure 3:**
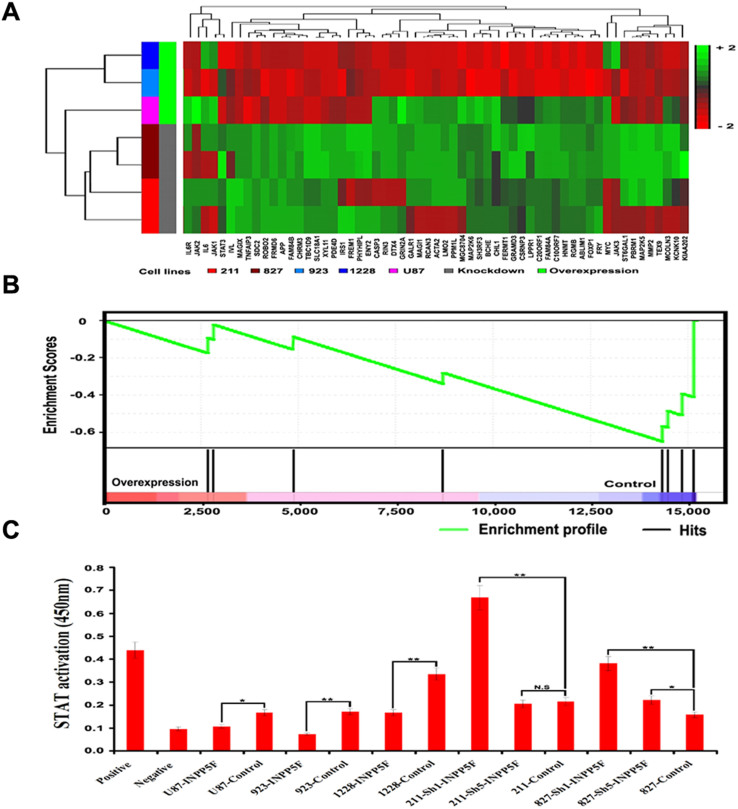
INPP5F regulates STAT3 pathway. (A) Hierarchical clustering heatmap of 59 INPP5F signature genes. The signature genes were derived by contrasting experimental control to the corresponding glioblastoma cell lines with either INPP5F overexpression (GSC1228, GCS923, and U87) or knockdown (GSC211 and GSC827). Green color in heatmap indicates increased expression and red color indicates decreased expression in cells with INPP5F overexpression and knockdown, respectively. The vertical bars represent cell lines. (B) Gene set enrichment analysis of STAT3 signaling pathway in INPP5F overexpressing1228 GSCs, compared with control. The green curve represents running enrichment score profile and the lower panel refers to the gene hits in STAT3 signaling pathway along the rank in ordered expression datasets. The normalized enrichment score for this analysis is -1.97 and the p-value is 0.015. (C) STAT3 activity analysis assay. Nuclear fractions were used for detection of STAT3 activity and observed in wavelength of 450 mn. Data show that the STAT3 activation was inhibited by INPP5F overexpression but enhanced by INPP5F knockdown as pair comparison, (NS: no significant; * p<0.05; ** p<0.001; compared to control). Error bars represent standard deviation (S.D., experiment performed in triplicates).

Furthermore, we performed mRNA expression microarray to see which genes are regulated by INPP5F, and analyzed the data with PARTEK program. The results showed that there are 59 genes were significantly regulated by INPP5F in both overexpressing and knockdown cells, compared to controls (Figure 3A). These data were also analyzed with GSEA (Gene Set Enrichment Analysis) and revealed that STAT3 pathway is strongly correlated with INPP5F expression (Figure 3B). To gain more insight into the effects of INPP5F on STAT3, we analyzed STAT3 activity with nuclear fraction. Consistent with the GSEA results, glioblstoma cells that overexpressed INPP5F showed a reduced STAT3 activation while the cells with INPP5F knockdown exhibited increased STAT3 activation (Figure 3C). These data demonstrate that INPP5F regulates STAT3 activation and STAT3 pathway related genes.

### INPP5F interacts with STAT3 directly, and inhibits STAT3 phosphorylation and nuclear translocation

**Figure 4:**
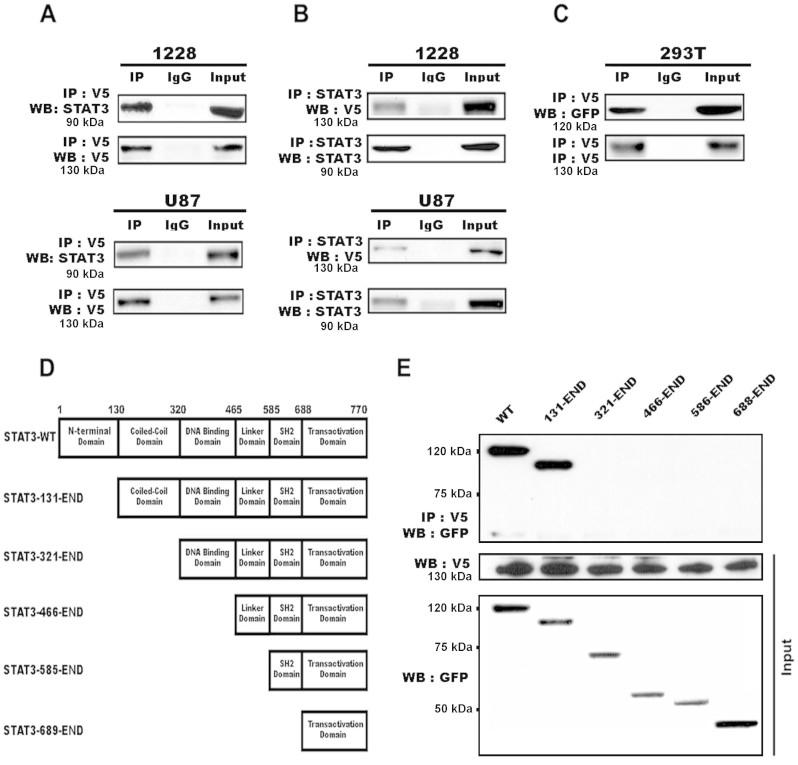
Immunoprecipitation (IP) analysis for INPP5F interaction with STAT3. (A) and (B) INPP5F overexpressing cells were lysed and immunoprecipitated with anti-V5 for INPP5F and anti-STAT3. Immunoblot analysis represents that INPP5F interacts with STAT3 in both cell lines with overexpressed INPP5F. (C) IP was performed with anti-V5 for INPP5F and immunoblot analysis represents that dominant negative STAT3 (Y705F) interacts with INPP5F in 293T cells. (D) Schematic diagrams of the structural domains and deletion mutants of STAT3. (E) Serial mutants of STAT3 and INPP5F were co-transfected in 293T cells and immuneprecipitated with anti-V5 for INPP5F. Immunoblot analysis represents that INPP5F interacted with full-length and deleted N-terminal domain of STAT3. Anti-GFP is used for STAT3 (Y705F) and its serial mutants, and anti-V5 for INPP5F.

INPP5F has been reported to regulate PIP3, AKT and GSK3 by dephosphorylations^3^,^4^,^5^, and we show here that INPP5F regulates STAT3 activation and its pathway (Figure 3). To identify proteins that interact with INPP5F, we performed co-immunoprecipitation (Co-IP) experiments using anti-V5 with INPP5F overexpressing cells, and found that INPP5F co-precipitated with STAT3 in glioblastoma cell lysates (Figure 4A). In addition, Co-IP using an anti-STAT3 antibody in the same lysates demonstrated that STAT3 co-precipitated with INPP5F (Figure 4B), indicating a direct interaction between INPP5F and STAT3. Next, we investigated protein interaction between mutant STAT3 at phosphorylation site and INPP5F, using STAT3-Y705F-GFP and INPP5F-V5 co-transfected 293T cells. As shown in Figure 4C, INPP5F also interacted with mutant STAT3 (Y705F), suggesting that INPP5F and STAT3 interaction may not be affected by phosphorylation in transactivation domain of STAT3. Based on the finding that INPP5F interacts with both wild type and dominant mutant STAT3, we further studied INPP5F binding domain of STAT3 using a series of STAT3 mutants (Figure 4D). It is identified that INPP5F interacts with full length and N-terminal domain deleted STAT3 (131-End) but not others (Figure 4E). These data demonstrate that INPP5F directly interact with coiled-coil domain of STAT3.

**Figure 5:**
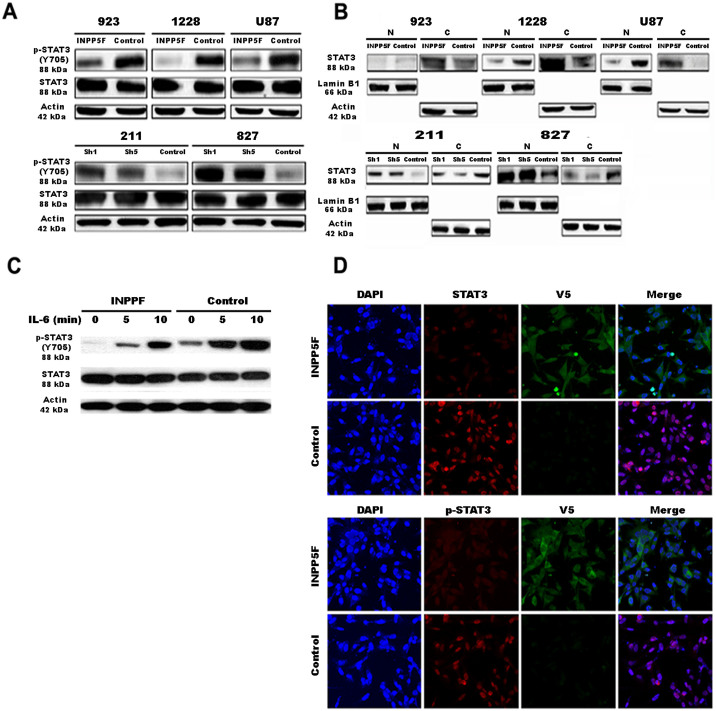
INPP5F inhibits STAT3 phosphorylation and nuclear translocation. (A) Immunoblot analysis shows that INPP5F overexpression in glioblastoma cells (GSC923, GSC1228, and U87) inhibits phosphorylation of STAT3 (Y705) and INPP5F knockdown (GSC211 and GSC827) enhances its phosphorylation. (B) Immunoblot analysis of nuclear [N] and cytosol [C] fractions reveals that overexpression of INPP5F inhibits STAT3 nuclear translocation while INPP5F knockdown enhances the nuclear translocation of STAT3. (C) Control and INPP5F overexpressing GSC923 cells were treated with IL-6 (50 ng/ml) for 5 and 10 min, and immunoblot was performed with anti-p-STAT3 (Y705). Immunoblot analysis shows that INPP5F inhibits IL-6 induced phosphorylation of STAT3. (D) Confocal microscopy analysis verified that overexpression of INPP5F decreases STAT3 phosphorylation and nuclear translocation compared to control.

Phosphorylation of STAT3 by several signals regulates the dimerization of STAT3 and these dimeric STAT3 translocate to the nucleus, where it binds to consensus STAT3 binding sequences within the promoter region of target genes and thereby activates their transcription^16^,^17^. To see whether STAT3 activity is regulated by its interaction with INPP5F, we analyzed the phosphorylation and localization of STAT3. As shown in Figure 5, INPP5F overexpressing cells exhibited a decreased STAT3 phosphorylation and an inhibition of STAT3 translocation into nucleus, while increased STAT3 phosphorylation and enhanced STAT3 translocation appeared in INPP5F knockdown cells (Figure 5A and 5B). The inhibitory effect of INPP5F on STAT3 phosphorylation was further demonstrated in INPP5F overexpressing GSC923 cells after treated with interleukin-6 (IL-6) stimulation (Figure 5C). Additionally, confocal microscopy analysis showed that INPP5F overexpression significantly inhibited STAT3 phosphorylation and its nuclear translocation (Figure 5D and Supplementary Figure S1). These data demonstrate that INPP5F inhibits STAT3 activity through INPP5F - STAT3 interaction and inhibition of STAT3 phosphorylation and translocation.

### INPP5F suppresses gliomagenesis and increases survival *in vivo*

**Figure 6:**
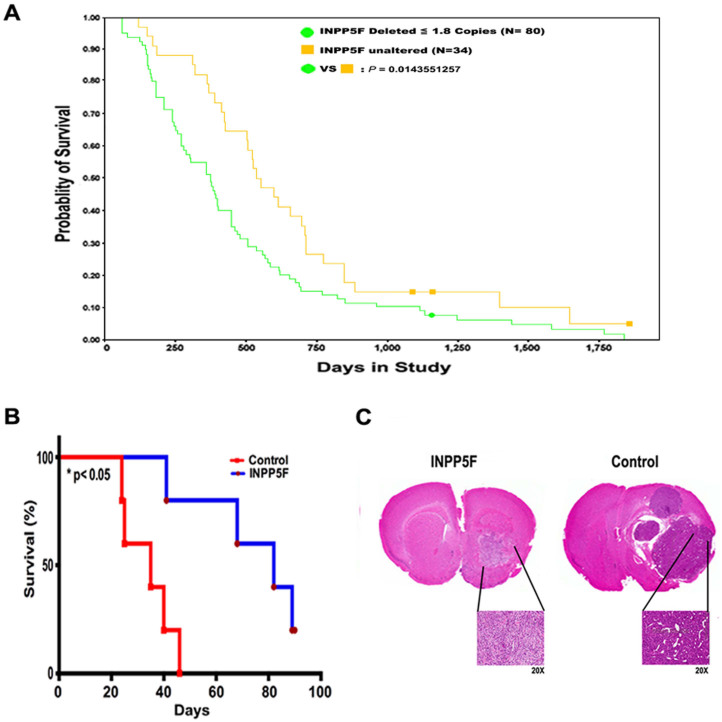
Correlation of INPP5F expression with gliomagenesis and survival. (A) *INPP5F* expression is correlated with GBM patient survival when glioma specimens are segregated in terms of copy number status using REMBRANDT database. Deletion of INPP5F correlated with poor patient survival, compared to that with unaltered INPP5F. (B) INPP5F overexpressing U87 cells intracranial injected mice show suppressed tumorigenesis compared to control U87 injected mice. Kaplan–Meier survival curves show a median survival time of 85.5 days for mice injected with INPP5F overexpressing U87 cells and 39.5 days for controls (log rank test, *p<0.05). (C) Intracranial tumor histopathological examination. Tumor sections were stained with hematoxylin and eosin (H&E) and examined for evidence of tumor mass at low and high magnification (upper and lower panels, respectively, of each pair). Compared to controls, mice injected with INPP5F overexpressing U87 cells show decreased tumor size and aggressiveness.

To further investigate the tumor suppressor effect of INPP5F in gliomas, we analyzed the INPP5F gene expression and glioma patient survival data contained in the REMBRANDT dataset. As shown in Figure 6A, glioblastoma patients with *INPP5F* deletion (<1.8 copies, 80 cases) have a short survival, significantly different from that with unaltered *INPP5F* (34 cases), p = 0.0144. Similar results observed in patients with all types of gliomas; Cases with *INPP5F* deletion (n = 103) showed a significant lower survival rate than that with *INPP5F* unaltered (n = 69), (Supplementary Figure S2, p = 2.78 × 10^−6^). Although the retrospective data cannot determine whether INPP5F could be an independent predictor of survival, these data that INPP5F loss in glioma patients is inversely correlated with survival do suggest that the expression of INPP5F might be a positive prognostic factor for glioma patients.

Finally, to evaluate the effect of INPP5F on glioma pathogenesis, INPP5F overexpressing U87 cells were injected into the periventricular cortex of adult female severe combined immunodeficiency (SCID) mice. Consistent with patients survival, mice inoculated with INPP5F overexpressing U87 cells lived significantly longer than control U87 inoculated animals (Figure 6B, median survival is 85.5 days for INPP5F and 39.5 days for control, respectively, p <0.05), with a smaller and more localized tumor observed histologically compared to control (Figure 6C). The data strongly supports that INPP5F contributes to inhibition of gliomagenesis as a tumor suppressor.

## Discussion

In this study, we analyzed TCGA and REMBRANDT databases and identified that *INPP5F* located in chromosome 10q26.11 is one of the highly altered glioma-related genes. The expression of INPP5F is significantly suppressed in glioblastomas and all types of gliomas, which correlated with decreased survival rates in glioma patients, suggesting that INPP5F might be a tumor suppressor in glioma pathogenesis.

Glioblastoma represents one of the most lethal human cancers with frequent biological relevant alterations. Among these, the RTK/PI3K/AKT pathway is one of the three critical signaling pathways that drive glioma growth^16^. PI3K-AKT pathway is deregulated in a wide spectrum of human cancers, and the gain or loss of function mutations of several components of the pathway lead to neoplastic transformation^18^. Downstream of PI3K by degradation of PI (3,4,5)P3 can be mediated by two different types of phosphatases. PTEN dephosphorylates the 3-position of PI (3,4,5)P3 to produce PI(4,5)P2, whereas SHIP (SH2-containing inositol-5-phosphatase) family phosphatases dephosphorylate the 5-position to produce PI(3,4)P2^19^,^20^. On the other hand, INPP5F, unlike the SHIP family phosphatases, can degrade both PI (3, 4, 5) P3 and PI (4, 5) P2 by removing the 5′ phosphate from inositol ring^3^ with Sac domain but not SH2-domain. Based on the fact that INPP5F regulates PI3K-AKT pathway by dephosphorylation and is significantly deregulated in gliomas, we hypothesized that INPP5F might be one of the critical factors in gliomagenesis by regulating PI3K-AKT and its related signaling pathways, such as STAT3^21^.

In the present study, we demonstrate that INPP5F strongly inhibits STAT3 activation by interaction with its coiled-coil domain and inhibits its phosphorylation, by which plays essential role in maintaining glioblastoma cells' phenotype and tumorigenic potential. STAT3, as a member of the STAT transcription factors family, is important in central nervous system development, reprogramming to ground state pluripotency, and essential for maintenance of embryonic stem (ES) cell biology, as well as glioblastoma tumorigenesis^22^,^23^. All STAT proteins are built up similarly, consisting of six conserved domains: a N-terminal domain followed by a coiled-coil domain, a DNA-binding domain, a linker domain, a SH2 domain and finally a transactivation domain. The phosphorylation of a specific tyrosine residue near the C-terminus is essential for STAT activation. Activated by cytokines and growth factors, STAT3 is phosphorylated on a single tyrosine residue at position 705, dimerizes, and accumulates in the nucleus to induce target gene expression, by which prominently contributes to cellular transformation and tumor maintenance, particularly in glioblastomas^24^,^25^,^26^. Sherry and colleagues reported that STAT3 is required for maintenances of multipotency in GSCs^27^. STAT3 transcriptional activity is regulated by PIAS (Protein inhibitors of activated STAT) proteins interaction with DNA binding via their N-terminal domains^28^, and its phosphorylation is regulated by several protein tyrosine phosphatases such as PTPRD (Receptor-type tyrosine-protein phosphatase delta) for inhibition^29^, or by kinases such as BMX (bone marrow X-linked kinase) for activation^30^,^31^.

Phosphorylation at tyrosine 705 is essential for STAT3 activation, and STAT3 Y705 point mutant (STAT3-YF) should be inactive. Using the STAT3-YF mutant we identified that INPP5F interacts with STAT3 directly and such an interaction is STAT3 phosphorylation site independent (Figure 4), the findings led us to analyze which domain of STAT3 is able to interact with INPP5F. The coiled -coil domain of STAT3 is essential not only for binding with SH2 domain-mediated receptor and EZH2, one of the Polycomb group (PcG) proteins, but also for its activation via phosphorylation^32^,^33^. Consistent with these references, we demonstrated that INPP5F directly interacts with the coiled-coil domain of STAT3 by IP experiments with a series of STAT3 mutants (Figure 4C and 4D). Furthermore, we demonstrated that INPP5F regulated STAT3 phosphorylation, translocation and activation (Figures 3C and 5). These data reveal that INPP5F interacts with STAT3 and regulates its activity in glioblastomas, suggesting that INPP5F is one of the novel phosphatases involved in STAT3 pathway. When analyzed the correlation between INPP5F in clinical samples and survivals of glioma patients, we found that consistent our experimental data, INPP5F expression is highly correlated with patient survivals (Figures 6A and Supplementary Figure S2), and these clinical relevance is supported by *in vivo* experiments (Figure 6B).

Taken together, for the first time, our study identified a novel role of INPP5F as a tumor suppressor in glioma tumorgenicity. *INPP5F* deletion is correlated with a decreased survival rate for glioma patients, and overexpressed INPP5F suppresses glioblastoma cell proliferation, GSCs' self-renewal, and gliomagenesis through inhibition of STAT3 pathway. In addition, as a number of STAT3 pathway related inhibitors are now in clinical trials, and loss of INPP5F expression may provide a marker for selecting patients who will respond to these drugs.

## Methods

### Reagents

Antibodies against to INPP5F (H00022876-B01P) were purchased from Novus Biologicals, Littleton, CO; Stat3 (sc-7179), p-Stat3 (sc-8059), GFP (sc-8334) and Actin (sc-8432) from Santa Cruz Biotechnology, Dallas, TX; V5 (A01724) from GenScript, Piscataway, NJ; Lamin B1 (33-2000) from Invitrogen (Life Technologies, Carlsbad, CA). Recombinant IL-6 was purchased from R&D System. The plasmids pLenti6/UbC/V5-DEST, pDEST47-DEST and ViraPower mix were purchased from Life Technologies; and INPP5F ShRNAs were from Open Biosystems (GE Dharmacon, Lafayette, CO).

### GSCs and glioblastoma cell lines culture

Glioblastoma stem cells (GSCs) were cultured in NBE media consisting of neurobasal medium, with N2 and B27 supplements (Life Technologies), and human recombinant bFGF and EGF (25 ng/ml each; R&D Systems, Minneapolis, MN) as previous described^34^. Human glioblastoma cell line U87 cells and human embryonic kidney (HEK) 293T cells were obtained from the American Type Culture Collection (ATCC; Manassas, VA) and cultured in Dulbecco's modified Eagle medium (DMEM) containing 5% fetal bovine serum (FBS), 1% L-glutathionine, and 1% penicillin–streptomycin (all from Life Technologies).

### Cloning and vector preparation

INPP5F (F: GCCACCATGGAGCTCTTCCAAGCCAAG GAC and R:GCCACCAATCTGAATTATCCGTGTCTGGCATTGTATAAAC) and STAT3 (F: GCCACCATGGCCCAATGGAATCAGCTACAGCAGC; 131F: GCCACCATGCCCAC AGCAGCCGTGGTGACG, 321F: GCCACCATGTTTGTGGTGGAGCGGCAGCCCTGCA TGCCC, 466F: GCCACCATGAACATCTGTCAGATGCCAAATGCC, 586F: GCCACCAT GGGCTTTATCAGTAAGGAGCG, 689F: GCCACCATGCCAGAGAGCCAGGAGCATCC TGAAGC and R: GCCACCCATGGGGGAGGTAGCG CACTCCGAGG) genes were inserted into Gateway vector pCR8/GW/TOPO and subsequently into pLenti6/UbC/V5-DEST. Site-directed mutagenesis was performed to generate STAT3 Y705F, using a Quick Change Site-Directed Mutagenesis kit (Stratagene, Agilent, Santa Clara, CA) according to the manufacturer's instructions. To change a base, STAT3 entry clone was used as a template. Primers (F: CCAGGTAGCGCTGCCCCATTC CTGAAGACCAAGTTTATC and R: GATAAACTTGGTCTTCAGGAATGGGGCAGC GCTACCTGG) were used. The nucleotide sequences of the mutated plasmids were confirmed by DNA sequencing. Lentivirus was produced in 293T cells with packaging mix (ViraPower Lentiviral Expression Systems, Life Technologies). Plasmids were transfected with Lipofectamine and Plus reagent (Life Technologies). After 3 hrs, media were changed to that with 5% FBS. Virus-laden supernatants were collected at 24, 48 and 72 hrs. The supernatant was filtered and concentrated by ultracentrifugation, and viral titer was determined by serial dilution.

### Cell proliferation, limiting dilution, and colony formation assays

Cultured glioblastoma cells were dissociated into single-cell suspensions. For proliferation assay, GSCs (923, 1228, 211, and 827) and U87 cells were plated into 6-well plates with 2 × 10^5^ cells per well and incubated at 37°C for 2 and 5 days. At the time of quantification, cells in each well were counted using Vi-CELL XR (BCM). For the limiting dilution assay, GSC cells were plated into 96-well plates at various seeding densities (2, 5, and 10 cells per well) and incubated at 37°C for up to 2 weeks. Each well was then examined for spheres formation. Soft agar colony formation assay was performed using standard protocol with a minor modification. Briefly, low-melting-point agar (Difco) was melted and mixed with DMEM containing serum medium at a 1:1 ratio to make a supporting bottom layer (1%) in a 2 ml/well (6-well plate). The bottom agar layer was allowed to solidify at room temperature for 20 min. The top layer containing 0.4% agar (2 ml/well) was prepared by mixing stock agar solutions with 100,000 cells in DMEM containing serum medium, and then laid on top of the supporting agar layer. Cell colonies were allowed to form at 37°C for 2–3 weeks. Experiments were performed at least in triplicates for each condition, and colonies in each well were counted.

### Immunoblot and immunoprecipitation analysis

Cells were collected and lysed in M2 buffer (20 mM Tris [pH 7], 0.5% NP40, 250 mM NaCl, 3 mM EDTA, 3 mM EGTA, 2 mM DTT, 0.5 mM PMSF, 20 mM β-glycerol phosphate, 1 mM sodium vanadate, and 1 tablet of protease cocktail). Immunoprecipitations were performed with cell lysates using Immunoprecipitation Kit –Dynabeads Protein G (10007D, Life technologies), according to the manufacturer's instruction. Cell lysates were separated by SDS-PAGE and analyzed by immunoblot. The proteins were visualized by enhanced chemiluminescence (ECL) according to the manufacturer's instruction (Pierce).

### Nuclear fraction and STAT3 activity analysis

Nuclear fractions were prepared using Nuclear Extract Kit (Active Motif, 40010) and the STAT3 activity analysis assay was performed using TransAM STAT3 kit (Active Motif, 45196) according to the manufacturer's instructions. Absorbance (450 nm) with a reference wavelength of 655 nm was detected by using a plate leader (POLARstar OPTIMA, BMG Labtech).

### Confocal microscopy analysis

Cells cultured in chamber slides (177402, Nalgene Nunc International) were fixed with 4% paraformaldehyde fixative (PFA) for 30 min at 4°C, permeabilized with 0.5% Triton X-100 in PBS, and blocked with 1% BSA. The cells were then stained with primary antibodies followed by Alexa Fluor-conjugated secondary antibodies (Life Technologies), and examined under confocal microscope (LSM700, Zeiss).

### TCGA (The Cancer Genome Atlas) and REMBRANDT (REpository of Molecular BRAin Neoplasia DaTa) data analysis

For expression analysis, gene expression array data of 389 glioblastomas and non-brain tumors generated using Affymetrix HG_U133A platform was downloaded from TCGA data portal (https://tcga-data.nci.nih.gov/tcga/tcgaHome2.jsp). The .CEL files were normalized and detection calls were generated using MAS5 algorithm in R ver. 2.12. The *INPP5F* expression data across all the patients were extracted and plot was created using Prism 6. *INPP5F* expression was also analyzed with REMBRANDT dataset of 577 brain tumors. For copy number analysis, 447 glioblastoma gene centered copy number data were downloaded from TCGA data portal. The copy number data of INPP5F and its neighboring genes BAG3 and MCMBP on chromosome 10 were extracted and the heatmap of these genes were created in MATLAB R2009b.

### mRNA expression microarray and data analysis

The mRNAs of four GSC lines, 211, 827, 923, 1228 and one established glioblastoma cell line U87, with either *INPP5F* overexpressing or knockdown, as well as their controls were extracted using TRIZOL (Life Technologies), purified using RNeasy Mini Kit (Qiagen), and then profiled to HG_U133 plus2 GeneChip Arrays according to the manufactory's instruction (Affymetrix). All .CEL files were normalized using MAS5 method and probe sets with only absent calls across all the samples were removed prior to data analysis. The fold changes of 1.2 were used for deriving INPP5F signatures. The hierarchical cluster analysis was performed using Partek software 6.6 (Partek Inc.) for Euclidean distance with complete linkage method. Gene set enrichment analysis was done using GSEA application (Subramanian et al., 2005). The enrichment scores were calculated by walking down the ordered list and the statistical significance of nominal *p* values of the enrichment scores were estimated using Kolmogorov-Smirnov statistics by constructing a cumulative null distribution with 1,000 permutations.

### Intracranial glioblastoma model, histology and H&E staining

An intracranial orthotopic model was utilized for evaluation of glioblastoma cells tumorigenicity according to an approved animal study proposal by NCI-ACUC. Cells were resuspended in 5 μl of HBSS and injected stereotactically into the striatum of female SCID mice (6–8 week age) using a stereotactic device (coordinates, 2 mm anterior and 2 mm lateral from bregma, and 2.5 mm depth from the dura). Following injection, animals were monitored daily for the development of neurological deficits. For histology examination, mice were euthanized by perfusion with 4% PFA under anesthesia, and brains were dissected, followed by overnight post-fixation in 4% PFA at 4°C. The 5 µm paraffin serial sections were prepared and stained with hematoxylin and eosin (Histo Serve, Inc). Images were captured using a Leica DM1400B microsystem and Leica FW4000 version 1.2.1.

### Statistical Analysis

Student's t test was used for data analysis and a *p* value ≤ 0.05 was considered significant. All values are shown as mean ± standard deviation (SD). Kaplan-Meier survival analysis was performed in Prism 4.0 software.

## Author Contributions

H.S.K. conceived and designed the experiments; H.S.K., S.A. and H.S. carried out the experiments; and H.S.K., A.L. and W.Z. analysed the data. H.S.K., A.L. and W.Z. wrote the manuscript, and H.S.K., A.L., S.A., H.S. and W.Z. discussed the results and commented on the manuscript. All authors read and approved the final manuscript.

## Supplementary Material

Supplementary Information Inositol Polyphosphate-5-Phosphatase F (INPP5F) inhibits STAT3 activity and suppresses gliomas tumorigenicity
