# Supplementary material for: Inositol Polyphosphate-5-Phosphatase F (INPP5F) inhibits STAT3 activity and suppresses gliomas tumorigenicity
Source: Sci Rep. 2014 Dec 5;4:7330. doi: 10.1038/srep07330 (PMC4256660; doi:10.1038/srep07330)

Inositol Polyphosphate-5-Phosphatase F (INPP5F) inhibits STAT3 activity and suppresses gliomas tumorigenicity

Hong Sug Kim[1,*], Aiguo Li[1], Susie Ahn[1], Hua Song[1], Wei Zhang[1,*]

[1]Neuro-Oncology Branch, National Cancer Institute and National Institutes of Neurological Disorders & Stroke, National Institutes of Health, Bethesda, MD 20892, USA

*Co-Correspondence: khongsug@gmail.com, zhangwe@mail.nih.gov

Short title: INPP5F suppresses STAT3 and gliomagenesis

Key words: INPP5F, gliomas, STAT3 signaling

No potential conflicts of interest were disclosed.

SUPPLEMENTARY FIGURE LEGENDS

Figure S1. Conforcal microscopy analysis. GSCs were dissociated and seeded poly-ornithine coated cover slip contained 24 well plates and analyzed with confocal microscope (Zeiss). Both INPP5F overexpressed GSCs (923 and 1228) showed decreased STAT3 phosphorylation and translocation compared with their controls.

Figure S2. Kaplan-Meijer analysis of glioma patient survival associated with INPP5F copy number status. INPP5F expression is correlated with patient survivals when all types of glioma patient specimens are segregated based on their INPP5F copy number status using the REMBRANDT database. Deletion of *INPP5F* correlated with poor patient survival compared to that with unaltered *INPP5F* in REMBRANDT.

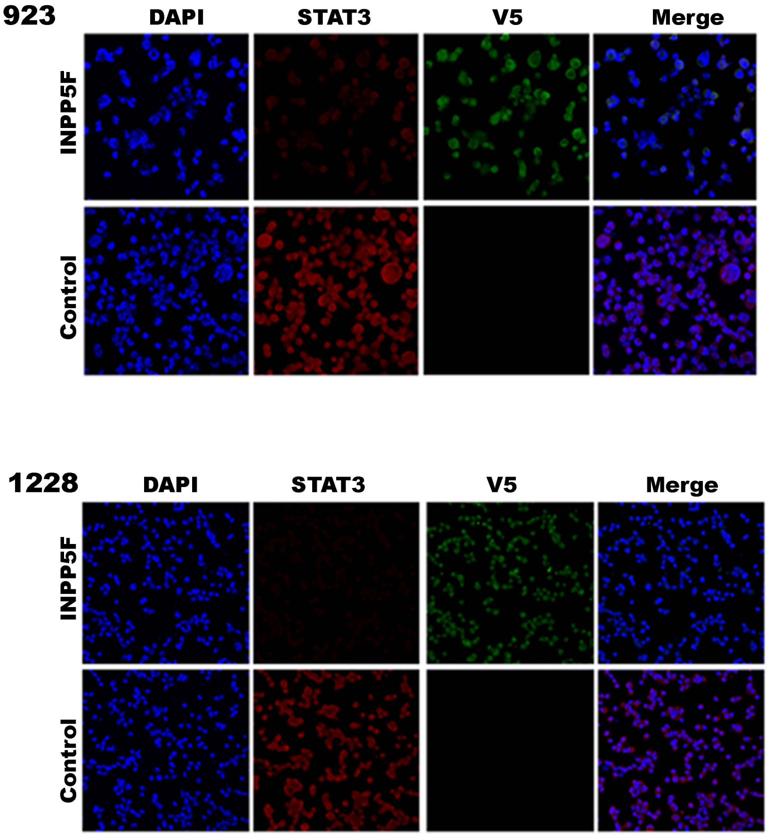
923
DAPI
STAT3
V5
Merge
INPP5F
Control
1228
DAPI
STAT3
V5
Merge
INPP5F
Control

Supplementary Fig. S2

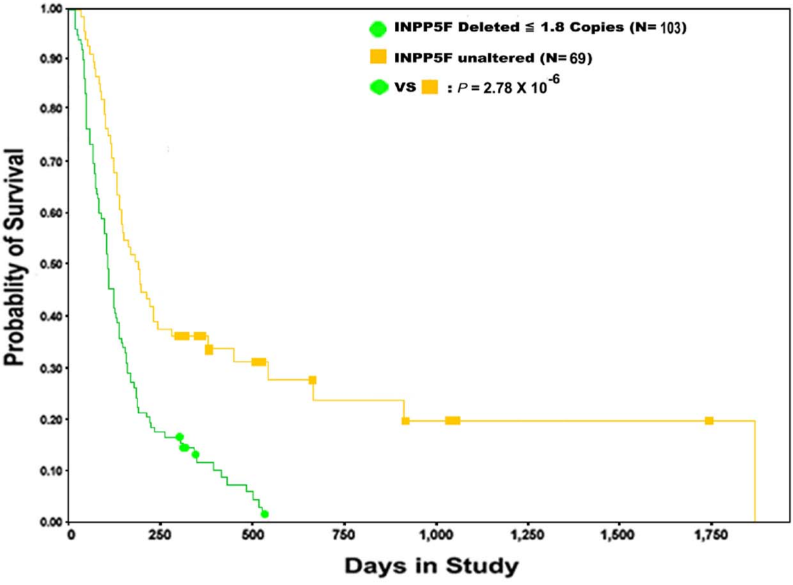

Supplement: Supplementary Information — Inositol Polyphosphate-5-Phosphatase F (INPP5F) inhibits STAT3 activity and suppresses gliomas tumorigenicity [file srep07330-s1.pdf]
